# Editorial: Pathogenic mechanisms in neurodevelopmental disorders: advances in cellular models and multi-omics approaches

**DOI:** 10.3389/fcell.2023.1296885

**Published:** 2023-10-06

**Authors:** R. Hollstein, A. Peron, K. S. Wendt, I. Parenti

**Affiliations:** ^1^ Institute of Human Genetics, University of Bonn and University Hospital Bonn, Bonn, Germany; ^2^ Medical Genetics, Meyer Children’s Hospital IRCCS, Florence, Italy; ^3^ Department of Experimental and Clinical Biomedical Sciences “Mario Serio”, Università Degli Studi Di Firenze, Florence, Italy; ^4^ Department of Cell Biology, Erasmus MC, Rotterdam, Netherlands; ^5^ Institute of Human Genetics, University Hospital Essen, University Duisburg-Essen, Essen, Germany

**Keywords:** neurodevelopmental disorders, omics technologies, MAVE, animal models, VUS

Omics technologies have triggered a transformation in the field of clinical genetics and molecular biology. The evaluation of the whole genome in terms of genomic variations, transcriptome, epigenome and regulome (transcription factor binding, enhancer activity), also down to the single-cell level, has provided unprecedented insights for our comprehension of human health and disease complexity.

These omics approaches have been particularly instrumental in the field of genetics, especially for the understanding of neurodevelopmental disorders (NDDs) (Ristori et al., 2020; Di Paolo et al., 2021; Yousefi et al., 2021; Khodosevich and Sellgren, 2023). NDDs encompass brain-related conditions with a wide range of clinical signs, including intellectual disability, developmental delay, craniofacial dysmorphism, and behavioral anomalies (Parenti et al., 2020). Given their prevalence and lifelong impact, NDDs pose a significant challenge for affected individuals, their families, and the healthcare system, emphasizing the importance of advancing our knowledge and developing effective interventions. The molecular causes of NDDs have long remained elusive, leaving numerous patients without diagnosis and appropriate treatment. The introduction of next-generation sequencing (NGS) technologies has enabled rapid and cost-effective sequencing of gene panels, exomes, and genomes. This has dramatically accelerated the diagnostic process and provided answers to families navigating diagnostic odysseys. However, with an average diagnostic yield of 31%–53% for exome sequencing (Bruel et al., 2020), it appears evident that relying solely on one omics technology may not necessarily lead to a molecular diagnosis. Three studies in this Research Topic (Colin et al., Colin et al., and McConkey et al.) highlight the value of integrating multi-omics approaches to solve challenging diagnostic cases with rare genetic diseases. The findings of Colin et al. and McConkey et al. additionally emphasize the significance of genomic DNA methylation studies in combination with sequencing technologies to achieve a molecular diagnosis.

Besides improving the diagnostic success rate, omics technologies offer valuable support for genotype-first approaches. Such strategies consist in assembling patients’ cohorts based on their genomic variants rather than their phenotypic features. When combined with an in-depth phenotyping, the genotype-first approach enables a less biased and therefore more comprehensive understanding of the phenotypic range associated with different genetic alterations in a given gene. In their studies, Parenti et al. and Kampmeier et al. utilize the genotype-first strategy to refine the clinical spectrum of NDDs linked to specific genes (*SYN1* and *PHIP*, respectively) and emphasize the importance of timely molecular diagnosis and genetic counseling for appropriate patient management.

Despite the indisputable advances, the employment of omics technologies comes along with new challenges, like the increasing number of detected variants of uncertain significance (VUS). In this context, cellular models, such as patient-derived induced pluripotent stem cells (iPSCs) and organoids as well as various animal models can indeed facilitate the interpretation of the clinical significance of these variants and the identification of the underlying mechanisms of NDDs. As an example, Schmidt et al. employed deep sequencing analyses and performed expression studies using organoids from patient-derived iPSCs to characterize *STAG2* mosaicism and to reveal the essential role of *STAG2* in early brain development. Ahmed et al. shed light on the specific cellular and molecular alterations in cholinergic interneurons and their potential role in the pathogenesis of autism spectrum disorder through the investigation of developmental alterations of striatal interneurons in the Cntnap2 knockout mouse model. The review by Di Fede et al. further focuses on animal models that mimic human genetic conditions, highlighting the shared abnormalities and clinical manifestations between these models and human patients.

Further, Artificial intelligence (AI)-based methods offer valuable tools for the analysis of omics data and interpretation of VUSs at both the phenotypic and molecular levels (Gupta et al., 2022). For instance, Kampmeier et al. took advantage of the AI-based GestaltMatcher database (Hsieh et al., 2022) to categorize patients with variants in the *PHIP* gene based on their facial features and illustrated that *PHIP* patients can be distinguished from healthy individuals or individuals with other syndromes. Furthermore, several AI-based softwares that rely on bioinformatic predictions and clinical features are currently available for the prioritization of variants.

On the other side, the growing amount of data generated with omics technologies also raises new questions:

(i) Which strategies can aid the interpretation of VUSs? The identification of VUSs still leaves patients without genetic diagnoses and optimal clinical care. Assessing the functional impact of these variants individually using cellular or animal models is both resource-intensive and time-consuming. Consequently, only a fraction of genetic variants has been functionally characterized in the last decades. A promising method to improve VUS interpretation emerged with the development of Multiplexed Assays of Variant Effects (MAVEs). These assays enable the simultaneous assessment of thousands of variants within a single experiment, yielding variant effect maps for entire genes. These maps can additionally be used to train AI-based variant effect predictors. International collaborations like the Variant Effect Atlas Alliance aim to maximize the utility of genomics for diagnosing and treating diseases by providing and enhancing MAVE related resources and tools such as MaveDB (Esposito et al., 2019; Fowler et al., 2023).
(ii) Which omics technologies should become part of the routine diagnostic process? NGS is currently the most employed diagnostic method. However, the content of our Research Topic attests how variants could be missed when only a single omics technology is applied. Whether or not the combination of two or more technologies will represent a time- and cost-effective option as a first-tier analysis still needs to be determined (Wojcik et al., 2023), but the results of first multi omics-derived studies are promising (Lunke et al., 2023).
(iii) Can we translate the new knowledge into therapies? Omics technologies bear the potential to shed light on the pathomechanism responsible for the disease-onset and on the molecules that are altered in the disease-state, which could conceivably represent future therapeutic targets or biomarkers.

In summary, the integration of omics technologies and the adoption of genotype-first approaches, as shown in the studies of this Research Topic, are enabling substantial progress in the diagnosis as well as in the clinical and molecular characterization of NDDs. Addressing the aforementioned questions could offer substantial benefits to patients in terms of enhanced diagnostic precision, development of personalized treatment approaches, and better management.
